# Exploration of cerebral hemodynamic pathways through which large artery function affects neurovascular coupling in young women

**DOI:** 10.3389/fcvm.2022.914439

**Published:** 2022-08-12

**Authors:** Burak T. Cilhoroz, Jacob P. DeBlois, Wesley K. Lefferts, Allison P. Keller, Patricia Pagan Lassalle, Michelle L. Meyer, Lee Stoner, Kevin S. Heffernan

**Affiliations:** ^1^Department of Exercise Science, Syracuse University, Syracuse, NY, United States; ^2^Department of Kinesiology, Iowa State University, Ames, IA, United States; ^3^Department of Exercise and Sport Science, University of North Carolina, Chapel Hill, NC, United States; ^4^Department of Epidemiology, Gilling's School of Public Health, University of North Carolina at Chapel Hill, Chapel Hill, NC, United States; ^5^Department of Emergency Medicine, School of Medicine, University of North Carolina at Chapel Hill, Chapel Hill, NC, United States

**Keywords:** cerebral pulsatility, cognitive function, vascular stiffness, blood pressure, neurovascular coupling

## Abstract

**Background:**

The interactions between large artery function and neurovascular coupling (NVC) are emerging as important contributors to cognitive health. Women are disproportionally affected by Alzheimer's disease and related dementia later in life. Understanding large artery correlates of NVC in young women may help with preservation of cognitive health with advancing age.

**Purpose:**

To explore the association between large artery function, NVC and cognitive performance in young women.

**Methods:**

Vascular measurements were made in 61 women (21 ± 4 yrs) at rest and during a cognitive challenge (Stroop task). Transcranial Doppler was used to measure left middle cerebral artery (MCA) maximum velocity (V_max_), mean velocity (V_mean_), and pulsatility index (PI). NVC was determined as MCA blood velocity reactivity to the Stroop task. Large artery function was determined using carotid-femoral pulse wave velocity (cfPWV) as a proxy measure of aortic stiffness and carotid ultrasound-derived measures of compliance and reactivity (diameter change to the Stroop task). Cognitive function was assessed separately using a computerized neurocognitive battery that included appraisal of response speed, executive function, information processing efficiency, memory, attention/concentration, and impulsivity.

**Results:**

MCA V_max_ reactivity was positively associated with executive function (*β* = 0.26, 95% CI 0.01–0.10); MCA V_mean_ reactivity was negatively associated with response speed (*β* = −0.33, 95% CI −0.19 to −0.02) and positively with memory score (*β* = 0.28, 95% CI 0.01–0.19). MCA PI reactivity was negatively associated with attention performance (*β* = −0.29, 95% CI −14.9 to −1.0). Path analyses identified significant paths (p < 0.05) between carotid compliance and carotid diameter reactivity to select domains of cognitive function through MCA reactivity.

**Conclusions:**

NVC was associated with cognitive function in young women. Carotid artery function assessed as carotid compliance and carotid reactivity may contribute to optimal NVC in young women through increased blood flow delivery and reduced blood flow pulsatility.

## Introduction

Women are disproportionally affected by Alzheimer's disease and related dementia (ADRD) with advancing age (1) and rates of later life cognitive decline may be faster in women compared to men (2). Optimal cognitive function is dependent upon the vascular response to cognitive engagement (3); that is, a reactive cerebrovasculature ensures rapid redirection of cerebral blood flow (CBF) supply to regions of increased neuronal demand (3). The matching of cerebral supply to neuronal metabolic demand is known as neurovascular coupling (NVC). Impaired NVC contributes to development of cognitive decline and may precipitate the development of ADRD (4, 5). Thus, exploration of determinants of NVC in young women may offer insight into sex-specific mechanisms governing preservation of cognitive reserve across the lifespan (6, 7).

NVC is directly dependent on large artery structure and function. The aorta and carotid arteries are inherently elastic large arteries, serving important roles as hemodynamic buffers. As such, the stiffening of these large arteries affects not only the absolute amount of blood flow delivered to the brain during cognitive engagement *via* the cerebral circuit, but the manner in which that flow is delivered (8). Specifically, large artery stiffness begets high pulsatile hemodynamic transmission into the microvasculature of the brain (9, 10). As the brain operates at low vascular impedance, impaired pulsatile damping to the cerebral microvasculature inflicts cerebrovascular damage, dysfunction, and corresponding cognitive decline (9, 10). There are prominent sex differences in large artery stiffness and cerebral pulsatility, with women experiencing greater increases in large artery stiffness and cerebral pulsatility, and reduced pulsatile damping, across the lifespan compared to men (11, 12). There is currently a knowledge gap regarding the influence of large artery stiffness and cerebral pulsatility on NVC and, in turn, cognitive performance in young women.

Therefore, the aims of this exploratory study were to examine: (1) the associations of large artery structure and function with NVC in young women; and (2) potential paths from large artery stiffness to cognitive function through NVC using path analysis. We hypothesized that (1) large artery stiffness would be associated with NVC in young women; and (2) significant paths would be identified between large artery stiffness, NVC, and cognitive function in young women. Findings may provide insight into the role of large artery structure and function as regulators of NVC and cognitive function in young women, offering new potential targets for preservation of cognitive healthspan and prevention of cognitive decline later in life.

## Methods

### Participants

Participants for this study included young women (*n* = 61) between 18 and 35 years of age that were recruited from the local university and surrounding community. All women were free from cardiorespiratory and cerebrovascular diseases, metabolic diseases, renal diseases, and mild cognitive impairment (determined *via* the Montreal Cognitive Assessment). None of the participants used antihypertensive medication. Prior to study enrollment, all participants provided written informed consent and this study was approved by the Syracuse University Institutional Review Board.

### Study design and overview

Participants completed this cross-sectional study following two visits to the Human Performance Laboratory. Prior to the first visit, participants were asked to fast for 3 h. The first visit consisted of screening for mild cognitive impairment with the Montreal Cognitive Assessment (MOCA score < 26), anthropometric assessment, and familiarization with all vascular testing. Upon completing the first visit, participants were provided with an accelerometer to objectively assess physical activity levels. The second visit was scheduled ~9 days later and during the early follicular phase of the menstrual cycle. Participants arrived at the laboratory following a 12-h overnight fast. During the second visit, a finger-stick blood sample was used to obtain traditional cardiovascular and metabolic risk factors (i.e., fasting glucose and blood lipids). Participants then underwent vascular testing while in the supine position (baseline) and during two separate 3-min cognitive challenges. Vascular measures included peripheral and central blood pressure (BP), measures of aortic and carotid structure and function, and middle cerebral artery blood velocity. Following vascular-hemodynamic assessment, a 30-min computerized neurocognitive battery was administered. Participants then completed an online health history questionnaire. Both subjective factors (self-reported health history) and objective factors (body composition, body mass index [BMI], physical activity and traditional cardiovascular disease risk factors) were acquired to characterize general health status of participants.

#### Health screening

Participants completed a detailed online health history and sociodemographic questionnaire using REDCap (Vanderbilt University; Nashville, TN, USA).

#### Anthropometrics and body composition

Height was measured using a digital stadiometer (SONARIS, Detecto; Webb City, MO, USA). Weight was measured with a digital scale. Air displacement plethysmography (BodPod, Cosmed; Rome, Italy) was performed to determine body fat percentage. Healthy weight was operationally defined as a BMI between 18.5 and 24.9 kg/m^2^. Overweight was operationally defined as a BMI between 25 and 29.9 kg/m^2^ and obese class I was operationally defined as a BMI between 30 and 34.9 kg/m^2^.

#### Blood sampling

A finger stick blood sample was obtained to analyze fasting lipids and glucose using a validated point-of-care device (Cholestech LDX analyzer, Abbott Laboratories; Chicago, IL, USA).

#### Physical activity measurement

Participants wore an accelerometer (wGT3X, ActiGraph LLC; Pensacola, FL, USA) around the waist for 9 consecutive days to assess physical activity levels using cut-points developed by Freedson et al. (13). A complete day of accelerometer use was defined as at least 10 h of wear time while awake, which is consistent with the minimum set by the NHANES20, and a minimum of 4 days of wear data was necessary in order for participants to be included in data analysis. A cut point of 2020 activity counts/minute was used to determine the amount of time participants engaged in moderate-to-vigorous physical activity (MVPA) level.

#### Brachial blood pressure

Prior to any vascular testing, participants rested for 10-min in the supine position to ensure a stable hemodynamic state (i.e., two systolic and diastolic blood pressure measures within 5 mmHg). Brachial systolic blood pressure (SBP) and diastolic blood pressure (DBP) were measured at rest according to the American Heart Association (AHA) standards (14) using a validated, automated, oscillometer device (BP786N, OMRON Healthcare, Inc., Lake Forest, IL, USA).

#### Carotid thickness, stiffness, and compliance

Carotid thickness, stiffness and compliance were measured to assess large artery structure and function, respectively. Carotid artery intima-media thickness (IMT), stiffness, and compliance were assessed using ultrasound (ProSound α7, Aloka; Tokyo, Japan) and a 7.5–10.0 MHz linear-array probe positioned laterally ~1 cm below the left carotid bulb. Carotid IMT was analyzed from a 10-mm longitudinal view of the far wall of the artery (obtained parallel/horizontal to the probe) as the distance from the intima lumen interface to the media adventitial border using electronic calipers and semi-automatic inbuilt software. IMT measures were obtained in triplicate during diastole determined from simultaneous ECG (R-wave) gating and averaged for subsequent analyses. Carotid distension waves were then captured across the cardiac cycle using eTracking software from the same longitudinal image as used for IMT appraisal. Carotid beta-stiffness was calculated as ln(P_max_/P_min_)/[(D_max_-D_min_)/D_min_], where P denotes carotid pressure (measured simultaneously) and D corresponds to carotid diameter. Carotid compliance was calculated as the change in area calculated from diameter across the cardiac cycle relative to the change in pressure (Area_max_-Area_min_/ P_max_-P_min_). Max and min refer to maximum (systolic) and minimum (diastolic) values throughout the cardiac cycle, respectively, determined from simultaneous ECG gating. ECG was also used to determine heart rate (HR). Carotid pressure waveforms were captured simultaneously *via* applanation tonometry (SphygmoCor, AtCor Medical; Sydney, Australia) for a 10-s epoch and a composite waveform was produced. This composite waveform was calibrated to brachial DBP, and mean blood pressure (MBP, calculated as 1/3 SBP + 2/3 DBP) based on the assumed stability of these pressures throughout the systemic circulation to determine carotid SBP (cSBP) and used for aforementioned calculations of carotid stiffness and compliance.

#### Aortic stiffness

Carotid-femoral pulse wave velocity was used as proxy of aortic stiffness and taken as a measure of large artery function. Aortic and carotid stiffness are not redundant measures as each is a different vascular bed with differential associations with brain structure and function. Applanation tonometry (SphygmoCor, AtCor Medical; Sydney, Australia) was used to sequentially capture right carotid and right femoral pulse waveforms. The distance between each pulse site and the suprasternal notch was obtained using a tape measure, and the aortic path length was estimated by subtracting the carotid-suprasternal notch distance from the femoral-suprasternal notch distance. Carotid-femoral pulse wave velocity (cfPWV) was then calculated as the aortic path length divided by the time delay between ventricular contraction and the generation of the foot of the pressure waveform at each pulse site (15).

#### Cerebral blood velocity

We measured left middle cerebral artery (MCA) mean velocity (V_mean_), maximum velocity (V_max_), and pulsatility index (PI) to explore cerebral hemodynamic factors that reflect NVC. A 2 MHz transcranial Doppler probe (DWL Doppler Box-X, Compumedics; Singen, Germany) was placed at the left temporal window and signals were recorded at a depth of 45–60 mm. V_mean_ is conventionally used for the appraisal of NVC (16). However, PI may provide additional insight into NVC given its relation to arterial stiffness and distal/terminal cerebrovascular resistance. PI was calculated as V_max_-V_min_/V_mean_. Likewise, inclusion of V_max_ may complement V_mean_ and PI when appraising NVC. Specifically, V_max_ is: (1) strongly associated with systolic velocity in the aorta (17), (2) qualitatively similar to phase contrast magnetic resonance angiography when assessing cerebrovascular reactivity (18), and (3) less influenced by Doppler artifact than measures that include diastolic velocity (V_min_) (16).

#### Neurovascular coupling

Vascular-hemodynamic parameters were assessed during two separate computerized 3- min Stroop color-word tasks (E-Prime, Psychology Software Tools Inc., Sharpsburg PA) following 10-min of supine rest. The Stroop test consisted of congruent (lower cognitive load) and incongruent (higher cognitive load) tasks that were performed in a randomized order. The congruent task included words appearing in the same color as the target word itself (i.e., the word blue, written in blue), whereas the incongruent task comprised words appearing in a color incongruous with the target word (i.e., the word blue, written in red). During both tasks, participants selected one of the four response items that described the color of the target word as fast and as accurately as possible using a remote clicker, thus avoiding speaking and distortion of Doppler signals. Participant accuracy was adjusted to ~50% by manipulating response time intervals between trials to standardize cognitive load between participants and prevent task disengagement for specifically high or low performing individuals. The response time interval between trials would shorten by 300 ms for every three successive trials responded to correctly and vice-versa for incorrect answers. The response time ranged from 400 to 5,000 ms. For this study, we operationally defined NVC as change in MCA hemodynamics (V_mean_, V_max_, and PI) from rest to incongruent Stroop (19). The incongruent Stroop was used as our previous work has demonstrated this task to elicit a more robust hemodynamic response compared to the congruent Stroop (19, 20). Baseline MCA measures were calculated from the average of 4 consecutive 10-s epochs. Similarly, we averaged MCA measures from 4 consecutive 10-s epochs during Stroop tasks. MCA recordings were started ~30-s after initiation of each Stroop task to ensure a steady state was reached. Stroop tasks were separated by 4-min of quiet rest to ensure heart rate and blood pressure had returned to resting values (20). Additionally, we took change in carotid diameter (D_min_) from rest to incongruent Stroop as a measure of carotid artery reactivity (21).

#### Cognitive function battery

Following assessment of NVC, participants completed a 30-min WebNeuro computerized neuropsychological battery (Brain Resource WebNeuro, Total Brain; San Francisco, CA, USA) to test several domains of cognitive function. Participants completed tasks in the seated position with a standard mouse and keyboard. The content, convergent and divergent construct validity (22, 23) as well as test–retest reliability of WebNeuro have been established previously (24). The domains of cognitive function used in the current study included executive function, information processing efficiency, memory, attention/concentration, and impulsivity (23). These domains of cognition were tested by finger tapping, choice reaction time, memory recognition (verbal list-learning, immediate forced choice recall, and delayed forced choice recall), forward digit span, verbal interference, switching of attention, go/no-go (impulsivity), delayed memory recognition, sustained attention, and executive maze tasks.

WebNeuro data is linked to the Brain Resource International Database that takes age, self-reported biological sex, and education of participants into account when individual differences in scores are compared (23). Following each cognitive test, WebNeuro generates a summary of raw score metrics for all cognitive domains that are computed by creating the average of individual test scores (25). In order to standardize averaging across different units of measurement for each cognitive domain, raw scores were converted to norm-referenced z-scores, which were then averaged and reported as “standard ten” (STEN) scores (25). STEN scores range from 1 to 10 with a mean of 5.5 and standard deviation of 2 (26), indicating that a participant with a Mean ± SD of 5.5 ± 2 falls on the average score of the norm population. When we examined the relationship between cognitive function domains and hemodynamic responses, these STEN scores were used as representatives of cognitive outcomes in the statistical models.

### Statistical analyses

Assumptions of normality were assessed quantitatively using the Shapiro-Wilk test, and qualitatively *via* Q-Q plots and histograms. All data are reported as mean ± standard deviation for continuous data and as *n* (%) for categorical data. After testing normality of distribution, we first sought to confirm the efficacy of the Stroop task in eliciting a hemodynamic response in young women. To do so, a repeated measures analysis of variance was performed to determine changes in hemodynamic parameters from baseline to the congruent and incongruent Stroop tasks. Bonferroni-corrected alpha levels were utilized to limit type I error from multiple comparisons. Effect sizes were calculated for differences across time points as partial η^2^ (eta squared).

Next, we determined cerebral hemodynamic contributors (both resting and reactivity separately) to the different domains of cognitive function *via* block-wise linear regression. Separate models examined the contribution of baseline (resting) cerebral hemodynamics and cerebral reactivity on all domains of cognitive function, with cerebral reactivity calculated as described above as MCA V_mean_, V_max_, and PI during baseline (resting) subtracted from values measured during the incongruent Stroop task. Given the importance of perfusion pressure for cerebral blood flow, we entered MBP as a covariate in the first block for each model. When exploring resting cerebral factors as predictors of cognitive function, resting MBP was entered in the first block. When exploring cerebral reactivity factors as predictors of cognitive function, MBP reactivity was entered in the first block. MCA V_mean_, V_max_, and PI reactivity were then entered into a second block using the stepwise function.

**Figure 1:**
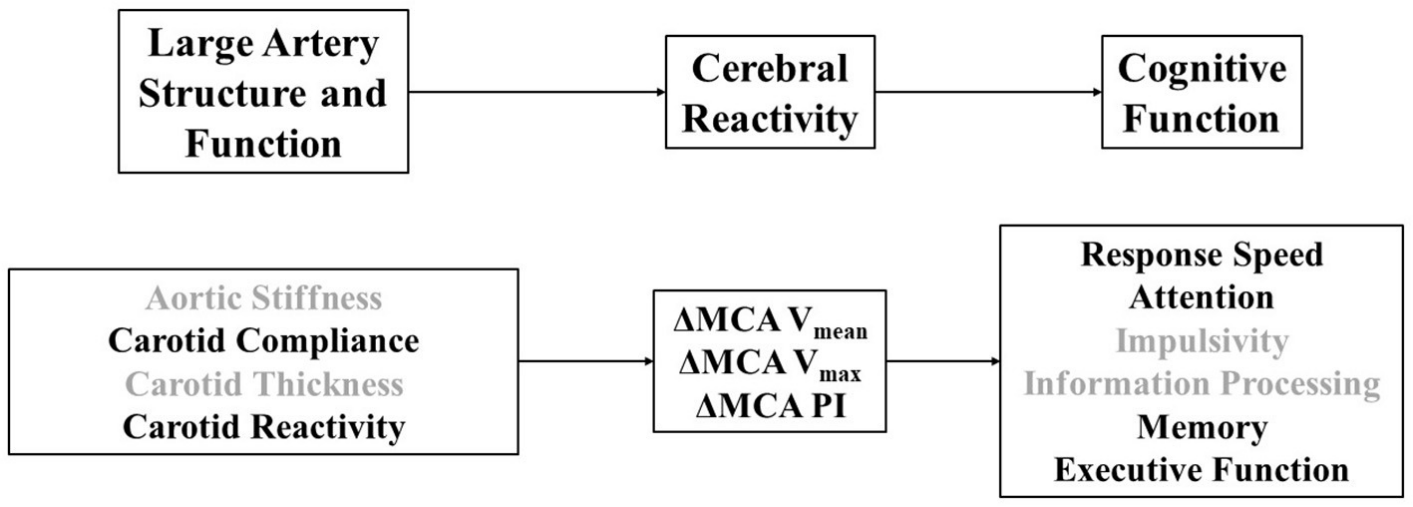
**(Top)** Theoretical model linking large artery structure (i.e., carotid intima-media thickness) and function (i.e., aortic stiffness, carotid stiffness, carotid compliance, carotid reactivity) to cerebral reactivity (MCA, middle cerebral artery; Vmean, mean blood velocity; Vmax, maximum blood velocity; PI, pulsatility index) and cognitive function. **(Bottom)** Operational definitions of specific physiological and cognitive constructs explored in this study that relate to our theoretical model. Darker font signifies variables that emerged as correlates across paths.

Path analysis was used to explore cerebral hemodynamic paths through which large artery structure and/or function might affect cognitive function (see Figure 1, Theoretical Model). To this end, we first examined strength of univariate associations of cfPWV, carotid beta-stiffness, carotid compliance, carotid reactivity and carotid IMT with cerebral reactivity measures using Pearson product-moment correlation coefficients to identify target measures of large artery structure and function for our subsequent paths associated with cognitive domains. We then constructed several paths and tested several model fit parameters, including: goodness of fit index (GFI), adjusted (for degrees of freedom) goodness of fit index (AGFI), normal fit index (NFI), relative fit index (RFI), comparative fit index (CFI), root mean square error of approximation (RMSEA), and Chi-square test. Fit was considered good if GFR, AGFI, NFI, RFI, and CFI were >0.95, RMSEA <0.05 and the *p*-value for the Chi-square test was > 0.05. For all other statistical tests, significance was established as *p* < 0.05. Data were analyzed using IBM SPSS Statistics and AMOS packages (v.27.0 Armonk, NY, USA).

## Results

Participants comprised a group a young women with intact cognitive function as evidenced by all MOCA scores being equal to or exceeding 26 (Table 1). Sixty-five percent of women had a healthy weight, 28% were considered overweight, and 7% were considered obese class I. According to accelerometry, 67% of participants were meeting or exceeding physical activity guideline recommendations. Women, on average, were completing 8,699 ± 2,297 steps per day. None self-reported a past diagnosis of CVD, diabetes, pulmonary disease, renal disease, hypertension, or dyslipidemia and none smoked, confirming our exclusion criteria. Five women reported a family history of CVD (1st degree relative/parent). None self-reported being amenorrheic and 29% were using oral contraceptives (mean duration 3.5 ± 3.0 years). All had fasting lipids and glucose within clinically acceptable ranges (Table 1).

**Table 1:** Descriptive characteristics of study participants (*n* = 61, Mean ± SD).

**Variables**	**Values**
Age (years)	21 ± 4
**Race (** ***n*** **, %)**	
Black/African American	17 (28)
White	35 (57)
Asian	2 (3)
American Indian	2 (3)
Other	5 (8)
**Ethnicity (** ***n*** **, %)**	
Hispanic	19 (31)
Non-Hispanic	42 (69)
**Education (** ***n*** **, %)**
High school	5 (8)
Some college	45 (74)
College	4 (7)
Graduate school	7 (11)
**Cardiometabolic Health Variables**	
Height (m)	1.64 ± 0.07
Weight (kg)	66.4 ± 10.4
Body mass index (kg/m^2^)	24.5 ± 3.6
Body fat (%)	29.7 ± 5.7
MoCA (score out of 30)	28 ± 1
MVPA (min/week)	365.6 ± 138.8
Oral contraceptive use (n, %)	18 (29)
Total cholesterol (mg/dL)	169 ± 34
HDL-C (mg/dL)	62 ± 17
LDL-C (mg/dL)	93 ± 32
Triglycerides (mg/dL)	67 ± 33
Glucose (mg/dL)	87 ± 6
Carotid IMT (mm)	0.39 ± 0.06

*MoCA, Montreal Cognitive Assessment; MVPA, moderate-to-vigorous physical activity; HDL-C, high-density lipoprotein; LDL-C, low-density lipoprotein; IMT, intima-media thickness*.

**Figure 2:**
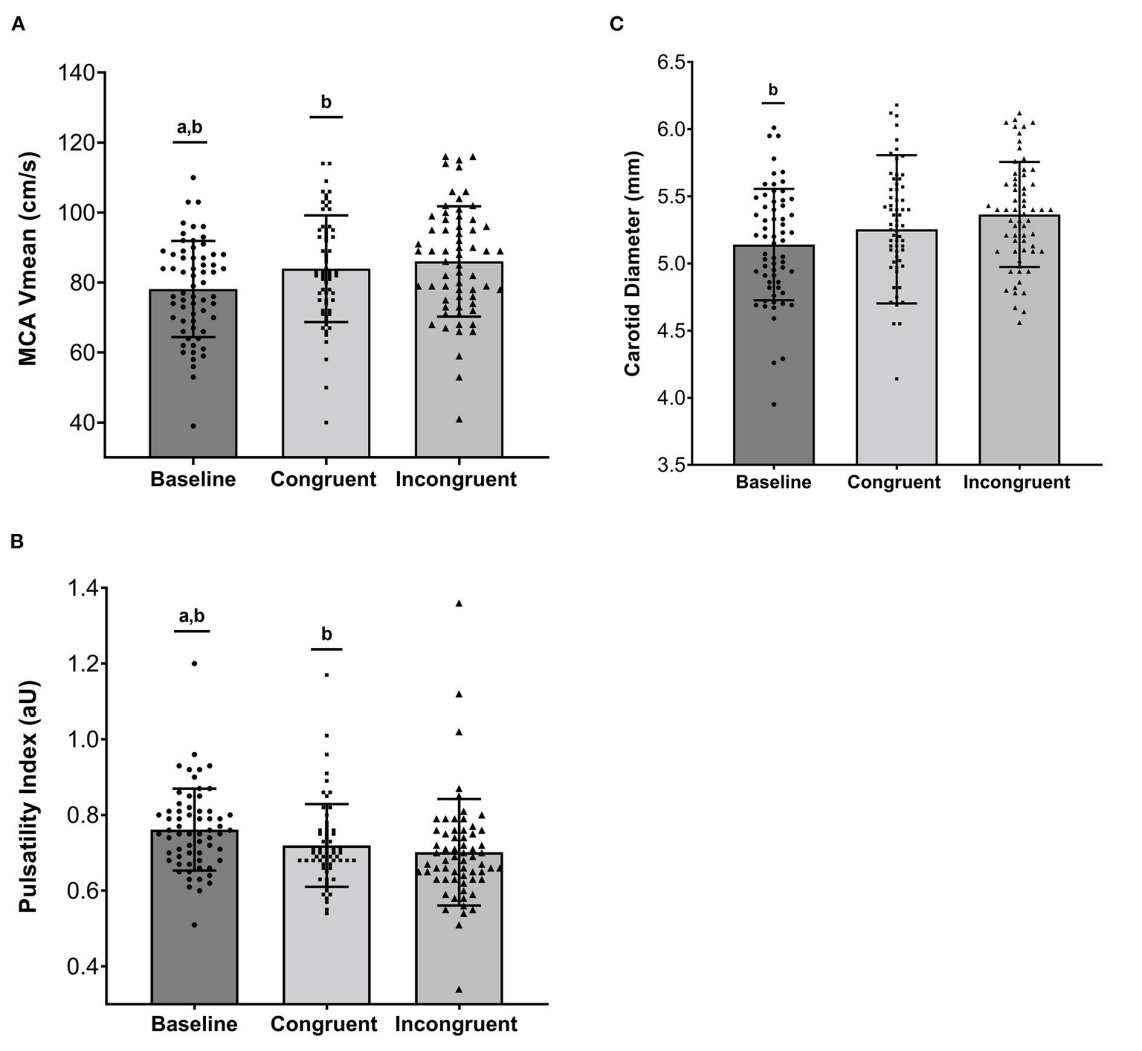
**(A)** Individual and mean ± standard deviation values for Middle Cerebral Artery Mean Blood Velocity (MCA Vmean, cm/s), **(B)** Middle Cerebral Artery Pulsatility Index (aU), and **(C)** Carotid Diameter (mm) across Baseline, Congruent Stroop, and Incongruent Stroop. ^a^Significantly different from Congruent (*p* < 0.05); ^b^Significantly different from Incongruent (*p* < 0.05).

There were stepwise increases in HR, SBP, DBP, and MBP from baseline to the congruent and incongruent Stroop (*p* < 0.001) confirming that an increase in cognitive load subsequently elicited an increase in hemodynamic load (Table 2). All changes in HR and BP had large effect sizes (partial η^2^ ≥ 0.24). Similarly, a stepwise increase in MCA blood velocities was observed from baseline to congruent and from congruent to incongruent Stroop tasks (Figure 2, *p* < 0.001). Large effect sizes were observed for increases in MCA V_max_ and V_mean_ across the study timepoints. MCA PI was reduced from baseline to congruent Stroop, and further reduced from congruent to incongruent Stroop (Figure 2, *p* < 0.001) also producing a large effect size. Finally, there were stepwise increases in cfPWV from baseline to congruent Stroop and again to incongruent Stroop (*p* = 0.003) as well as stepwise increases carotid artery diameter from baseline to congruent Stroop and again to incongruent Stroop (*p* = 0.001), with small to modest effect sizes noted. There was no change in carotid artery compliance or carotid beta-stiffness across conditions (*p* > 0.05).

**Table 2:** Changes in hemodynamics and vascular function during cognitive challenge (*n* = 61, Mean ± SD).

	**Baseline**	**Congruent stroop**	**Incongruent stroop**	**Significant time effect**	**Effect size^†^**
Heart rate (bpm)	60 ± 8^b^	63 ± 12	65 ± 9	**<0.001**	0.40
Systolic blood pressure (mmHg)	111 ± 9^a^^,^^b^	115 ± 13^b^	117 ± 11	**<0.001**	0.24
Diastolic blood pressure (mmHg)	72 ± 7^a^^,^^b^	75 ± 7^b^	77 ± 8	**<0.001**	0.35
Mean blood pressure (mmHg)	85 ± 7^a^^,^^b^	88 ± 8^b^	90 ± 9	**<0.001**	0.36
Carotid diastolic diameter (mm)	5.12 ± 0.42^b^	5.24 ± 0.55	5.35 ± 0.38	**<0.001**	0.14
Carotid compliance (mm^2^/kPa)	1.51 ± 0.29	1.50 ± 0.35	1.48 ± 0.39	0.772	0.01
Carotid beta-stiffness (aU)	3.3 ± 0.9	3.2 ± 1.0	3.3 ± 1.1	0.781	0.01
Carotid-femoral PWV (m/s)	5.4 ± 0.6	5.5 ± 0.8	5.6 ± 0.8	**0.003**	0.12
MCA max blood velocity (cm/s)	116 ± 16^a^^,^^b^	120 ± 18^b^	122 ± 17	**<0.001**	0.27
MCA mean blood velocity (cm/s)	80 ± 12^a^^,^^b^	85 ± 13^b^	87 ± 14	**<0.001**	0.41
MCA pulsatility index (aU)	0.75 ± 0.08^a^^,^^b^	0.72 ± 0.08^b^	0.70 ± 0.09	**<0.001**	0.22

a *Significantly different from Congruent (p < 0.05)*.

b *Significantly different from Incongruent (p < 0.05)*.

*PWV, pulse wave velocity; MCA, middle cerebral artery*.

† *Effect sizes were calculated for differences across time points as partial η^2^ (eta squared). Bold values indicate statistical significance (p < 0.05)*.

MCA reactivity to the incongruent Stroop task revealed significant associations with select domains of cognitive function, specifically response speed, memory, and executive function. Parsimonious models from stepwise regression are presented in Table 3 (variables that fell out of each model are not presented). Variance inflation factors for all models presented in Table 3 fell between 1.05 and 1.77 suggesting minimal concern for collinearity. Baseline (resting) cerebral measures (MCA V_max_, V_mean_, and PI), were not associated with any domain of cognitive function (*p* > 0.05 for all, Supplementary Table 1).

**Table 3:** Association between cerebral reactivity and select domains of cognitive function.

**Cerebral reactivity**	**Unstandardized *β***	**Standardized *β***	**t**	***p*-value**	**95% CI**	
					**Lower**	**Upper**
**Response speed**						
MBP reactivity	−0.02	−0.07	−0.53	0.60	−0.11	0.06
Vmean reactivity	−0.12	−0.33	−2.59	**0.012**	−0.19	−0.02
**Memory**						
MBP reactivity	−0.03	−0.07	−0.58	0.57	−0.11	0.06
Vmean reactivity	0.10	0.28	2.27	**0.027**	0.01	0.19
**Executive function**						
MBP reactivity	−0.01	−0.02	−0.13	0.89	−0.08	0.07
Vmax reactivity	0.05	0.26	2.06	**0.044**	0.01	0.10
**Attention**						
MBP reactivity	0.10	0.28	2.28	**0.027**	0.01	0.08
PI reactivity	−9.0	−0.29	−2.29	**0.026**	−14.9	−1.0

*MBP, mean blood pressure. Bold values indicate statistical significance (p < 0.05)*.

**Figure 3:**
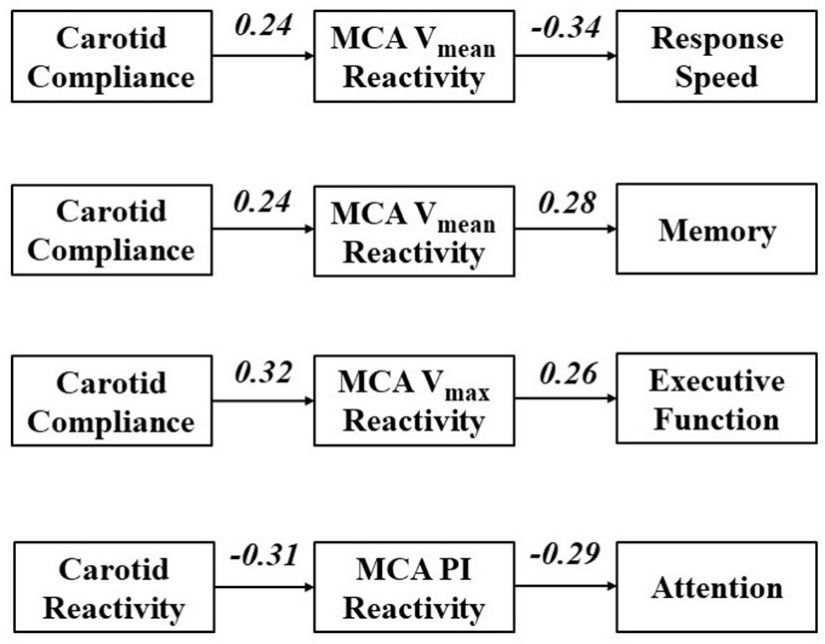
Path analyses exploring cross-sectional paths through which carotid artery function affects cerebral reactivity, in turn affecting domains of cognitive function. Values represent standardized regression weights.

Results for path analyses are displayed in Figure 3. Parameters were included in the model based on initial univariate correlations and a theoretical model that broadly conceptualizes large artery structure/function affecting cerebral hemodynamics, in turn affecting cognitive function (Figure 1). Univariate correlations between measures of vascular function and NVC are presented in Supplementary Table 1. First, we explored paths from carotid compliance and MCA V_mean_ reactivity to response speed and memory. The path from carotid compliance to MCA V_mean_ reactivity approached significance (*β* = 0.24, *p* = 0.052) while the path from MCA V_mean_ reactivity to response speed achieved significance (*β* = −0.34, *p* = 0.003). The model fit the data moderately well (GFI = 0.96, AGFI = 0.75, NFI = 0.75; RFI = 0.24, CFI = 0.765; RMSEA = 0.22; Chi-square = 3.975, *p* = 0.046). The path from MCA V_mean_ reactivity to memory also achieved significance (*β* = 0.28, *p* = 0.022). Goodness of fit was high for the path from carotid compliance to MCA V_mean_ reactivity to memory (GFI = 0.99, AGFI = 0.95, NFI = 0.92; RFI = 0.74, CFI = 1.00; RMSEA = 0.00; Chi-square = 0.813, *p* = 0.367). Next, we explored a path from carotid compliance to MCA V_max_ reactivity and executive function. Significant paths from carotid compliance to MCA V_max_ reactivity (*β* = 0.32, *p* = 0.008) and MCA V_max_ reactivity to executive function (*β* = 0.26, *p* = 0.038) were identified. The model fit the data well (GFI = 0.99, AGFI = 0.94, NFI = 0.93; RFI = 0.78, CFI = 1.00; RMSEA = 0.00; Chi-square = 0.857, *p* = 0.355). Finally, we explored a path from carotid reactivity to MCA PI reactivity and attention. Significant paths from carotid reactivity to MCA PI reactivity (*β* = −0.31, *p* = 0.013) and MCA PI reactivity to attention/concentration (*β* = −0.29, *p* = 0.021) were identified. The model fit the data well (GFI = 0.99, AGFI = 0.98, NFI = 0.98; RFI = 0.93, CFI = 1.00; RMSEA = 0.00; Chi-square = 0.265, *p* = 0.607).

## Discussion

We identified different cerebral pathways through which large artery function may affect select domains of cognitive function in young women. Our main findings can be broadly summarized as follows: (1) measures of carotid artery function appraised as carotid artery compliance and carotid reactivity are significant correlates of NVC in young women and (2) NVC is associated with response speed, memory, attention and executive function in young women. Taken together, our findings support an association between NVC and cognitive function in young women and suggest optimal NVC may depend on a compliant carotid artery effectively dilating during times of cognitive engagement to accommodate increased flow delivery, ensuring cerebrovascular perfusion with minimal pulsatility.

### CBF reactivity offers insight into cognitive function not captured by resting measures

Resting CBF, particularly hypoperfusion, has been shown to predict cognitive function in older adults (27, 28) with poor cerebrovascular function serving as an early marker of cognitive decline in older women (29). Interestingly, resting measures of CBF were not associated with cognitive function in young women in our study. We found that cerebral reactivity assessed during a cognitive challenge (our measure of NVC) had positive associations with the cognitive domains of response speed, memory, attention and executive function. Our results are consistent with previous work from Csipo et al. finding that increased cognitive workload is needed to evoke greater NVC responses in healthy young adults (30). Thus, in healthy women, increasing cognitive demand with a cognitive challenge like Stroop may stress cognitive reserves, revealing associations between cerebral perfusion and cognitive function not detected in a quiescent state. More research will be needed to unravel the clinical merit of studying NVC as studied herein as a potential biomarker of cerebrovascular health and cognitive performance.

### CBF PI reactivity is an important component of NVC

We observed that CBF pulsatility reactivity was negatively associated with the cognitive domain of attention in young women. Previous findings demonstrated a positive correlation between pulsatility and the severity of cognitive dysfunction in patients with dementia (4), mild cognitive impairment (31), AD (32), cerebral white matter disease (33), stroke, and early vascular dementia (34). The microcirculation in areas of the brain associated with long-term memory is especially vulnerable to pulsatile stress (as assessed by MCA PI) and is altered early in aging independent of demographic and cardiovascular covariates (35, 36). Our findings did not show a connection between pulsatility and memory scores, but it is important to note that attention and memory are intricately linked as attention is of vital importance for the encoding and recovery of long-term memory (37, 38). Additionally, a recent review reports that pulsatility and cognition associations eventually spread to other cognitive domains with increasing age (39). Chronic pulsatility is implicated as a detrimental hemodynamic factor contributing to cerebral microbleeds and ischemia over time (9, 10). In an acute setting, CBF pulsatility may affect oxygen delivery to the neurovascular unit, impacting neuronal function and thus cognitive performance (19). Our findings support the contention that pulsatility may be an important hemodynamic factor to measure when assessing NVC (40, 41). Longitudinal studies will be needed to determine if the association between acute changes CBF pulsatility during cognitive challenge and lower attention scores noted in our work could be indicative of compromised long-term memory later in life.

### Aortic stiffness is not associated with NVC in young women

Another noteworthy finding in the current study was that aortic stiffness was not associated with NVC in young women. Several studies in the literature indicate that vascular function as examined by aortic stiffness contributes to alterations in CBF and CBF pulsatility (42–44). Reasons for the discrepancy between our findings and others (42–44) are not clear but may be attributed to differences in age. Alterations in aortic stiffness demonstrate a gradual and incremental (non-linear) increase with advancing age in healthy individuals (45). However, women experience drastic increases in aortic stiffness at ~55–75 years of age compared to age-matched men, corresponding to the post-menopausal period (46). Parallel to substantial aortic stiffening, older women also have less pulsatile damping between the carotid and cerebral arteries compared with age-matched men, illustrating a higher transmission of pulsatile energy into the brain of older women (11, 12). This may be associated with dysfunction of the cerebral endothelial cells, a more permeable blood-brain barrier, neurovascular uncoupling, reduced CBF, and increased neuropathology (47). We suggest that the interaction of vascular aging and menopause-induced deterioration in arterial function might make the association between aortic stiffness and cognitive performance more prominent in later life. Overall, our findings suggest that aortic stiffness is not associated with cerebral reactivity and NVC in young women. Further research is needed to explore possible links between healthy vascular aging (i.e., low aortic stiffness across the lifespan) and cognitive reserve.

### Carotid artery function contributes to NVC in young women

While aortic stiffness was not associated with NVC in our study, our results showed that functional properties of the carotid artery were contributors to NVC. Functional vascular properties are those operationally defined as being actively modified over a short period of time. Such dynamic changes can be mediated by a variety of hemodynamic and autonomic factors affecting vascular smooth muscle cell (VSMC) tone and acute changes in interactions between VSMCs, elastin, collagen and the extracellular matrix impacting mechanical load bearing properties of the vessel wall. We noted an association between carotid compliance and carotid reactivity with CBF reactivity in young women such that greater carotid compliance and reactivity was associated with greater CBF reactivity and lower pulsatility, respectively. The change in carotid artery diameter during cognitive challenge has been suggested to be influenced by carotid endothelial function (21). This is important to note as endothelial dysfunction has been implicated in neurovascular uncoupling in older adults (6, 48). Moreover, the strength of association with NVC was slightly greater for carotid compliance vs. carotid stiffness. Compared to compliance, carotid stiffness parameters are thought to be less sensitive to the effects of blood pressure, and thus more closely approximate intrinsic vessel wall properties, however this belief has been challenged (49). Thus, some of the effect of carotid compliance on NVC may be pressure-mediated. Overall, our findings support a growing consensus that some cerebrovascular regulation occurs at the level of the carotid artery (50). The carotid artery is not a passive conduit but a dynamic contributor to NVC. We suggest that for optimal NVC, a compliant and reactive carotid artery dilates during cognitive engagement to ensure that increases in CBF occur without excessive increases in pulsatility.

###  Body fat is associated with carotid artery function in young women: Implications for NVC and cognitive function

Obesity is associated with structural and functional evidence of brain aging (51), impaired NVC (52) and reduced cognitive function in midlife (53). In our study, while measures of body composition were not associated with NVC or cognitive function, higher body fat percentage was associated with lower carotid compliance and attenuated carotid reactivity. Our findings are consistent with a broader literature noting the detrimental impact of higher body fat on arterial stiffness and vascular function (54, 55). It is reasonable to speculate that the impact of body fat on carotid function in young women may have a cumulative detrimental effect on NVC and cognitive function later in life. Body fat-mediated effects of inflammation may impact NVC *via* direct effects on the neurovascular unit (i.e., endothelial dysfunction, perivascular astrocyte senescence, microglia dysfunction—all collectively contributing to altered cerebral dilation and perfusion) (56–58). Body fat may also have indirect effects on the neurovascular unit *via* the mediating role of carotid artery function on cerebrovascular pulsatility. Parenthetically, we noted an inverse association between physical activity and body fat in young women. This finding may be taken to underscore the importance of physical activity on cerebrovascular health (59, 60). In general physical activity, exercise, maintaining high cardiorespiratory fitness and select dietary interventions (e.g., time-restricted feeding, cocoa) may all favorably target the neurovascular unit and be considered therapeutic lifestyle interventions to attenuate vascular aging, preserve NVC and maintain cognitive reserve across the lifespan (58, 61–63).

### Limitations and considerations

This study is subject to limitations. The cross-sectional design of our study does not allow us to establish causality or temporality of association. Hence, the results of correlations should be interpreted with caution. We performed the quantification of CBF through transcranial Doppler. Using transcranial Doppler for MCA blood velocity reactivity measurement has several advantages. Transcranial Doppler may be as accurate as computerized tomography and magnetic resonance angiography for the measurement of CBF in the basal cerebral arteries and their main branches including the MCAs (64). As the Doppler measures MCA blood velocity using the temporal window (the thinnest part of the skull in adults) (65), the potential interruption of ultrasound energy transmission from the Doppler due to bone thickness is also minimized. Nonetheless, transcranial Doppler has limitations for CBF reactivity measurement. The Doppler assumes that artery length, artery diameter, and blood viscosity are stable for velocity to be a substitute for CBF (66). Moreover, the Doppler signals can be influenced by the changes in the intracranial distal and extracranial proximal arteries (65). We examined unilateral MCA, thus, findings might not encompass the cognitive performance that could have been impacted by anterior, posterior and/or global CBF with bilateral measurement (67). Furthermore, the potential contribution of sex hormones and oral contraceptive use to vascular reactivity and cognitive function is unclear in our study as previous studies indicate estrogen- or progesterone-dependent changes in cerebrovascular hemodynamics among women (68, 69). Cognitive function, particularly executive function, may change during different phases of the menstrual cycle in women owing to the variations in the bioavailability of sex hormones (70, 71). We performed measurements during the early follicular phase of the menstrual cycle. Therefore, our findings may not capture a comprehensive picture of NVC in young women across the menstrual cycle with additional modulation by oral contraceptive use. Future studies that include men are needed to determine sex-specific effects of vascular function on NVC and cognitive function. Finally, although we included a diverse group of women in our study, we were underpowered to explore racial and ethnic variation in NVC and cognitive outcomes. Additional studies are warranted to explicitly explore the impact of race and ethnicity on vascular aging and NVC as potential mediators of racial and ethnic variation in cognitive aging.

## Conclusion

Our findings suggest that cerebral reactivity during cognitive challenge offers insight into NVC and cognitive performance in young women. Increased CBF reactivity and decreased cerebral pulsatility during acute cognitive challenge are related to higher cognitive performance. Additionally, carotid artery functional properties, namely carotid compliance and carotid reactivity, are associated with CBF reactivity. Thus, optimal NVC requires a compliant carotid artery that dilates during the cognitive challenge to ensure that increased flow delivery to the cerebrovasculature occurs with minimal pulsatility. A less compliant artery with poor dilatory capacity may not adequately buffer hemodynamic pulsatility, resulting in impaired NVC and reduced cognitive performance.

## Data availability statement

The raw data supporting the conclusions of this article will be made available by the authors, without undue reservation.

## Ethics statement

The studies involving human participants were reviewed and approved by Syracuse University IRB. The patients/participants provided their written informed consent to participate in this study.

## Author contributions

BC contributed to statistical analyses, results interpretation, and manuscript writing and reviewing. JD contributed to study design conceptualization, data collection, statistical analyses, results interpretation, and manuscript reviewing. WL and MM contributed to results interpretation and manuscript reviewing. AK contributed to data collection. PPL contributed to data collection and manuscript reviewing. LS contributed to statistical analyses, results interpretation, and manuscript reviewing. KH contributed to study design conceptualization, statistical analyses, results interpretation, manuscript writing and reviewing, and acquisition of funding. All authors contributed to the article and approved the submitted version.

## Funding

Funding for this study was provided by the National Institutes of Health – National Institute of Minority Health and Health Disparities (NIMHD R03MD011306).

## Conflict of interest

The authors declare that the research was conducted in the absence of any commercial or financial relationships that could be construed as a potential conflict of interest.

## Publisher's note

All claims expressed in this article are solely those of the authors and do not necessarily represent those of their affiliated organizations, or those of the publisher, the editors and the reviewers. Any product that may be evaluated in this article, or claim that may be made by its manufacturer, is not guaranteed or endorsed by the publisher.
