# Supplementary material for: Exploration of cerebral hemodynamic pathways through which large artery function affects neurovascular coupling in young women
Source: Front Cardiovasc Med. 2022 Aug 12;9:914439. doi: 10.3389/fcvm.2022.914439 (PMC9411931; doi:10.3389/fcvm.2022.914439)
Supplement: Supplementary file 1 [file Table_1.DOCX]

**Table 1 Supplement.** Correlations matrix between measures of vascular function and NVC

|  | Vmean Rest | Vmax Rest | PI Rest | Vmean Reactivity | Vmax Reactivity | PI Reactivity | cfPWV | Carotid Compliance | Carotid Reactivity | Response Speed | Memory | Executive Function | Attention | Body Fat |
| --- | --- | --- | --- | --- | --- | --- | --- | --- | --- | --- | --- | --- | --- | --- |
| Vmax Rest | **0.95** |  |  |  |  |  |  |  |  |  |  |  |  |  |
| PI Rest | **-0.21** | 0.07 |  |  |  |  |  |  |  |  |  |  |  |  |
| Vmean Reactivity | 0.03 | 0.01 | -0.08 |  |  |  |  |  |  |  |  |  |  |  |
| Vmax Reactivity | -0.09 | -0.14 | -0.11 | **0.61** |  |  |  |  |  |  |  |  |  |  |
| PI Reactivity | -0.16 | -0.19 | -0.12 | **-0.46** | 0.06 |  |  |  |  |  |  |  |  |  |
| cfPWV | 0.05 | 0.03 | -0.01 | 0.05 | -0.01 | 0.08 |  |  |  |  |  |  |  |  |
| Carotid Compliance | 0.17 | 0.19 | 0.04 | **0.24** | **0.32** | -0.13 | 0.07 |  |  |  |  |  |  |  |
| CCA Reactivity | -0.03 | -0.02 | 0.05 | 0.09 | 0.02 | **-0.31** | 0.02 | 0.04 |  |  |  |  |  |  |
| Response Speed | -0.05 | -0.03 | 0.16 | **-0.33** | -0.18 | **0.23** | -0.05 | 0.15 | -0.16 |  |  |  |  |  |
| Memory | 0.07 | 0.02 | -0.18 | **0.28** | **0.23** | -0.17 | 0.01 | 0.18 | 0.06 | 0.01 |  |  |  |  |
| Executive Function | 0.14 | 0.12 | 0.02 | 0.18 | **0.26** | 0.10 | 0.02 | 0.19 | 0.14 | **0.30** | **0.31** |  |  |  |
| Attention | -0.14 | -0.09 | 0.16 | **0.27** | 0.15 | **-0.29** | 0.02 | 0.01 | 0.03 | 0.04 | **0.32** | **0.48** |  |  |
| Body Fat | 0.01 | 0.01 | 0.01 | 0.04 | 0.06 | 0.08 | 0.19 | **-0.27** | **-0.24** | -0.08 | -0.01 | -0.15 | -0.09 |  |
| MVPA | 0.10 | 0.09 | -0.12 | -0.10 | -0.17 | **-0.22** | **-0.22** | 0.15 | 0.13 | 0.10 | 0.08 | 0.05 | 0.21 | **-0.35** |

**Bold** denotes significant correlation at p<0.05.

PI, pulsatility index; cfPWV, carotid-femoral pulse wave velocity; MVPA, moderate-vigorous physical activity
